# Strategies for conjugating iridium(III) anticancer complexes to targeting peptides via copper-free click chemistry

**DOI:** 10.1016/j.ica.2019.119396

**Published:** 2019-12-23

**Authors:** Wen-Ying Zhang, Samya Banerjee, Cinzia Imberti, Guy J. Clarkson, Qian Wang, Qian Zhong, Lawrence S. Young, Isolda Romero-Canelón, Musheng Zeng, Abraha Habtemariam, Peter J. Sadler

**Affiliations:** a Department of Chemistry, University of Warwick, Coventry CV4 7AL, UK; b State Key Laboratory of Oncology in South China, Collaborative Innovation Centre for Cancer Medicine, Sun Yat-Sen University Cancer Centre, Guangzhou 510060, China; c Medical School, University of Warwick, Coventry CV4 7AL, UK; d School of Pharmacy, Institute of Clinical Sciences, University of Birmingham, Birmingham B15 2TT, UK

**Keywords:** Organo-iridium, Anticancer, Azide-alkyne cycloaddition, Copper-free, Cyclic peptide

## Abstract

We report the synthesis and characterization of novel pentamethylcyclopentadienyl (Cp*) iridium(III) complexes [(Cp*)Ir(4-methyl-4′-carboxy-2,2′-bipyridine)Cl]PF6 (Ir-I), the product (Ir-II) from amide coupling of Ir-I to dibenzocyclooctyne-amine, and its conjugate (Ir-CP) with the cyclic nona-peptide c(CRWYDENAC). The familiar three-legged ‘piano-stool’ configuration for complex Ir-I was confirmed by its single crystal X-ray structure. Significantly, copper-free click strategy has been developed for site-specific conjugation of the parent complex Ir-I to the tumour targeting nona-cyclic peptide. The approach consisted of two steps: (i) the carboxylic acid group of the bipyridine ligand in complex Ir-I was first attached to an amine functionalized dibenzocyclooctyne group via amide formation to generate complex Ir-II; and (ii) the alkyne bond of dibenzocyclooctyne in complex Ir-II underwent a subsequent strain-promoted copper-free cycloaddition with the azide group of the modified peptide. Interestingly, while complex Ir-I was inactive towards A2780 human ovarian cancer cells, complex Ir-II exhibited moderate cytotoxic activity. Targeted complexes such as Ir-CP offer scope for enhanced activity and selectivity of this class of anticancer complexes.

## 1 Introduction

To reduce the toxic side effects and circumvent intrinsic or acquired resistance of the widely used clinical anticancer drug cisplatin [1–4],new transition-metal based anticancer agents are urgently needed [5–8]. Low-spin 5d^6^ organo-iridium(III) complexes have attracted wide attention [9,10]. Especially promising are half-sandwich iridium complexes [(η^5^-Cp*)Ir(XY)Cl]^+^ (where Cp* is pentamethylcyclopentadienyl and XY is a chelating ligand) which have emerged as potential next-generation anticancer agents with novel redox-mediated mechanisms of action to overcome platinum resistance [11,12]. The carbon-bound Cp* ligand forms highly stable iridium(III) complexes [13–15]. We have reported that phenyl or biphenyl substituents on the Cp* ring, or a switch of the chelating ligand from N^N’ to C^N mode can have pronounced effects on the anticancer activity of these complexes. Meanwhile, the iridium centre can catalyse hydride transfer from NADH to molecular oxygen, generating hydrogen peroxide in cancer cells to trigger cell death [11,16]. However, achieving selectivity toward tumour cells over normal cells for half-sandwich iridium complexes is still To reduce the toxic side effects and circumvent intrinsic or acquired challenging and worthy of further investigation [17–20].

Ruiz et al. have designed a half-sandwich Cp* iridium steroid hormone conjugate targeted to steroidal receptors, which displayed 6-fold and 2-fold greater potency than cisplatin and a non-steroidal analogue, respectively [21]. Recently, Liu et al. have further developed a series of half-sandwich iridium anticancer complexes with lysosome-targeting properties [22]. In addition, Perrier et al. have used a cyclic peptide—polymer nanotube to deliver a Cp* iridium anticancer complex, with remarkable selectivity toward cancer cells as well as higher activity toward human ovarian cancer cells compared to the free iridium complex [23].

Tumour-targeting peptides can also be used as vectors [24] and have the potential to provide alternative efficient therapeutic benefit over the drug on its own [25]. The sequence RWY (Arg-Trp-Tyr) within the cyclic peptide of sequence c(CRWYDENAC) has a high affinity and specificity for the integrin α6 receptor on the surface of nasopharyngeal carcinoma [26]. Conjugation of this peptide to the periphery of a Pt^IV^prodrug encapsulated in nanoparticles has resulted in a 100-fold increase in cytotoxicity over free cisplatin *in vitro* [26]. Furthermore, we have recently reported that photoactive Pt^IV^ prodrugs conjugated to this cyclic peptide exhibit higher photocytotoxicity and cellular Pt accumulation than the parent complex upon light irradiation [27].

However, it is well known that peptides with specific amino acid residues such as histidine, cysteine, tryptophan, and glutamic acid are natural chelating ligands for metal ions [28,29]. Conventional peptide conjugation methods suffer from poor site-specificity and hetero-geneous products related to the ratios of conjugation sites. To improve the site-specific conjugation as well as batch-to-batch reproducibility, “click” chemistry has proved to be a highly successful tool [30–32]. While the classic click Cu(I)-catalysed azide-alkyne cycloadditions have been a benchmark in many areas of recent synthetic chemistry, side reactions and toxicity of the copper catalyst often limit its utilization in biological applications [33,34]. By replacing terminal alkynes with strain-promoted cycloalkynes, copper-free azide-alkyne cycloadditions can be achieved under mild conditions without disrupting the function of the biomolecules, including selective derivatization of proteins, sugars, lipids, DNA and RNA [35]. The simplicity and orthogonality of strain-promoted azide-alkyne cycloadditions, have been widely used in a bioorthogonal fashion at the level of living cells as well as multicellular organisms [36–38].

Here we have synthesized a half-sandwich iridium complex [(Cp*)Ir (4-methyl-4′-carboxy-2,2′-bipyridine)Cl]PF_6_ **(Ir-I)** and its conjugate **(Ir-CP)** with a tumour-targeting cyclic peptide of sequence c(CRWYDENAC). The conjugation is achieved by an amide formation reaction first affording the precursor complex **Ir-II** for the subsequent strain-promoted copper-free azide-alkyne cycloaddition reaction between **Ir-II** and azide functionalised-cyclic peptide to generate the final **Ir-CP**. **Ir-I** and **Ir-II** are novel and have been characterized by ^1^H and ^13^C NMR, high resolution ESI-MS, HPLC and X-ray crystallography. The cytotoxicity of **Ir-I** and **Ir-II** has been screened against the human ovarian A2780 cancer cell line. The successful synthesis of the peptide conjugate **Ir-CP** has been verified by high resolution ESI-MS, HPLC, and UV–vis spectroscopy.

## 2 Experimental section

### 2.1 Materials

IrCl_3_ hydrate was purchased from Precious Metals Online. 1,2,3,4,5-Pentamethylcyclopentadiene, dibenzocyclooctyne-amine (DBCO-NH_2_),2.0 M oxalyl chloride, triethylamine (TEA), and analytical grade sodium chloride were purchased from Sigma-Aldrich. 4,4′-Dimethyl-2,2′-bipyridine was obtained from Carbosynth, and NH_4_PF_6_ from Alfa Aesar. Solvents of laboratory grade were used for synthesis without any further purification. HPLC grade water and acetonitrile used for RP-HPLC experiments with the additive, HPLC grade trifluoroacetic acid (TFA) were obtained from Fisher Scientific. Roswell Park Memorial Institute (RPMI-1640) medium, with 10% fetal bovine serum (FBS), and 1% v/v penicillin/streptomycin was used in the cellular experiments. The cyclic azide-functionalized peptide Azhx-c(CRWYDENAC) was purchased from Cambridge Research Biochemicals (Billingham, UK) with a purity of > 95% (HPLC/MS) with all L-configured amino acids in the sequence.

### 2.2 Analytical methods

^1^H,^1^H-^1^H COSY, and ^13^C APT NMR spectra were recorded on Bruker Avance III HD 400 MHz and Bruker Avance III HD 500 MHz spectrometers using the residual signal of the solvent as a chemical shift reference.^1^H NMR chemical shifts were internally referenced to (CHD_2_) OD (3.31 ppm) for methanol-*d*_4_. ^13^C NMR chemical shifts were internally referenced to CD_3_OD (49.15 ppm) for methanol-*d*_4_. The data were processed using Mestrenova and Topspin (version 2.1 Bruker UK Ltd.)

High resolution ESI-MS analysis was carried out using a Bruker MaXis plus Q-TOF mass spectrometer equipped with electrospray io-nisation source. The mass spectrometer was operated in electrospray positive ion mode with a scan range 50–2,400 *m/z*. Samples were prepared in methanol.

Electronic absorption spectra were recorded on a Varian Cary 300 UV–vis spectrophotometer in a 1 cm quartz cuvette and referenced to the solvent. The spectral scan was from 800 to 200 nm at ambient temperature and the bandwidth was 1.0 nm; the scan rate was set to 600 nm/min.

Analytical reversed-phase HPLC analyses were carried out on an Agilent ZORBAX Eclipse XDB-C18 column (250 × 4.6 mm, 5μm, flow rate: 1 mL/min) with mobile phases of 0.1% v/v TFA in H_2_O (solvent A) and 0.1% v/v TFA in CH_3_CN (solvent B). A linear gradient from 10% to 80% solvent B within 0–30 min was used with detection wavelength at 254 nm except for the free peptide (230 nm). LC-MS was carried out on a Bruker Amazon X with a scan range of *m/z* 50–2000 in positive mode connected online to an Agilent 1260 HPLC with the same detection wavelength as HPLC analyses.

### 2.3 Synthesis and characterization

4-Methyl-4′-carboxy-2,2′-bipyridine [39,40], and [(Cp*)Ir(μ-Cl)Cl]_2_[41] were synthesized and characterized following reported procedures.

**Ir-I**: [(Cp*)Ir(μ-Cl)Cl]2 (39.8 mg, 0.05 mmol) was dissolved in methanol (20 mL) followed by addition of the 4-methyl-4′-carboxy-2,2′-bipyridine ligand (21.4 mg, 0.10 mmol). The mixture was stirred at 313 K overnight. The final yellow solution was filtered, concentrated and NH_4_PF_6_ (163 mg, 1.0 mmol) was added to the solution. This solution was kept at 277 K overnight. The yellow precipitate was collected by filtration and dried under vacuum. Yield 66 mg, 91%; 1H NMR (400 MHz, d_4_-MeOD, 298 K): *δ* 9.11 (d, 1H, *J* = 5.8 Hz), 9.00 (s, 1H),8.81 (d, 1H, *J* = 5.8 Hz), 8.61 (s, 1H), 8.26 (d, 1H, *J* = 5.8 Hz), 7.71 (d,1H, *J* = 6.1 Hz), 2.70 (s, 3H), 1.72 (s, 15H); ^13^C NMR (125 MHz, d_4_-MeOD, 298 K): 165.79, 158.15, 155.99, 155.08, 154.10, 152.47,143.72, 131.21, 129.19, 126.65, 124.58, 91.28, 21.39, 8.67; high resolution ESI-MS *m/z* calcd for [(M−PF6−H)+Na]^+^ 599.1040, found 599.1038.

**Ir-II**: Complex **Ir-I** (26 mg, 0.036 mmol) was dissolved in anhydrous DCM (10 mL) in a two-neck round-bottomed flask to obtain a clear yellow solution. Oxalyl chloride (0.5 mL 2.0 M) was injected slowly under nitrogen into the solution which was cooled over an ice bath. A catalytic amount of anhydrous DMF was then added and the solution colour changed from yellow to red due to the carboxylic acid group of complex **Ir-I** being converted to the acyl chloride. The reaction mixture was further stirred for 2 h at 298 K and the solvent was taken off under vacuum to obtain a yellow residue. In a new two-neck flask, anhydrous DCM (10 mL), TEA (0.2 mL) and dibenzocyclooctyne-amine (DBCO-NH_2_) (10 mg, 0.036 mmol) were added. The acid chloride was resuspended in anhydrous DCM (8 mL) and added dropwise to the ice cooled mixture in the new flask within 0.5 h. The final mixture was stirred at 298 K for 4 h under nitrogen and dark conditions. The solvent was evaporated under vacuum and the crude solid obtained was purified on an Al_2_O_3_ column with MeOH/DCM (1/4 v/v). Yield 23 mg, yellow solid, 65%; ^1^H NMR (400 MHz, d_4_-MeOD, 298 K): *δ* 9.09 (d, 0.5H, *J* = 5.8 Hz), 9.00 (d, 0.5H, *J* = 5.9 Hz), 8.86 (d, 0.5H, *J* = 4.3 Hz), 8.83 (d, 0.5H, *J* = 4.2 Hz), 8.72 (s, 0.5H), 8.57 (s, 0.5H), 8.47 (s, 0.5H), 8.33 (s, 0.5H), 8.05 (d, 0.5H, *J* = 5.9 Hz), 7.86 (d, 0.5H, *J* = 5.8 Hz), 7.75–7.73 (m, 1H), 7.65 (d, 0.5H, *J* = 7.5 Hz), 7.62 (d, 0.5H, *J* = 7.6 Hz), 7.51–7.41 (m, 4H), 7.32 (t, 0.5H, *J* = 7.6 Hz); 7.20 (t, 0.5H, *J* = 7.4 Hz), 7.14 (t, 0.5H, *J* = 7.4 Hz), 6.72–6.86 (m, 1H), 6.75 (d, 0.5H, *J* = 7.5 Hz), 5.13 (d, 1H, *J* = 14.0 Hz), 3.66 (d, 1H, *J* = 13.7 Hz), 3.48 (m, 2H), 2.61–2.54 (m, 1H), 2.73 (s, 1.5H), 2.72 (s,1.5H), 2.47–2.39 (m, 1H), 1.73 (s, 15H); 13C NMR (125 MHz, d_4_-MeOD, 298 K): 173.35, 164.65, 156.07, 154.87, 153.72, 152.75, 152.58, 149.52, 145.82, 133.59, 131.27, 130.56, 130.22, 129.91, 129.07, 128.37, 127.97, 127.46, 126.47, 126.58, 126.53, 126.28, 124.19, 123.64, 122.76, 122.26, 115.62, 91.17, 38.22, 34.96, 21.55, 8.73; high resolution ESI-MS *m*/*z* calcd for [M – PF_6_]^+^ 835.2379, found 835.2383.

**Ir-CP:** Complex **Ir-II** (14.5 mg, 0.015 mmol) and azide-peptide Azhx-c(CRWYDENAC) (7.0 mg, 0.006 mmol) were dissolved in DMF (2 mL) to make a clear solution. The mixture was stirred under nitrogen and dark conditions at 298 K overnight.The solvent was then removed under vacuum and acetonitrile (2 mL) was added to wash the residue three times to obtain the final product. Yield 4 mg, light yellow solid, 30%; high resolution ESI-MS, *m*/*z* calcd for C_94_H_112_IrN_21_O_19_S_2_ [(**Ir-CP**)-Cl]^2+^ 1047.8755, found 1047.8739; calcd for C_94_H_113_IrN_21_O_19_S_2_ [(**Ir-CP**)-Cl+H]^3+^ 699.9194, found 698.9178.

### 2.4 X-ray crystallography

Single crystals of **Ir-I·MeOH** were grown from methanol/diethyl ether at room temperature. A suitable crystal was selected and mounted on a glass fibre with Fomblin oil and placed on a Rigaku Oxford Diffraction SuperNova diffractometer with a dual source (Cu at zero) equipped with an AtlasS2 CCD area detector. The crystal was kept at 150(2) K during data collection. Using Olex2 [42], the structure was solved with the ShelXT [43] structure solution program using Intrinsic Phasing and refined with the ShelXL [44] refinement package using Least Squares minimisation. X-ray crystallographic data for complex **IrI·MeOH** have been deposited in the Cambridge Crystallographic Data Centre under the accession number CCDC 1959197. X-ray crystallographic data in CIF format are available from the Cambridge Crystallographic Data Centre (http://www.ccdc.cam.ac.uk/).

### 2.5 Cell culture

A2780 human ovarian cancer cells were grown in Roswell Park Memorial Institute medium (RPMI-1640) supplemented with 10% (v/v) of fetal calf serum, 1% (v/v) of 2 mM glutamine, and 1% (v/v) penicillin/streptomycin. All cells were grown as adherent monolayers at 310 K with a 5% CO_2_ humidified atmosphere, and were passaged at ca. 80% confluency.

### 2.6 In vitro cell growth inhibition

Briefly, 5000 cells per well were seeded in 96-well plates. The cells were incubated in fresh drug-free medium at 310 K for 48 h before adding different concentrations of test compounds. Stock solutions of the complexes were prepared in DMSO (<0.5%)/cell culture medium. The drug exposure period was 24 h. After this, supernatants were removed by suction and each well was washed with 150 µL PBS once. The cells were allowed a further 72 h recovery in fresh medium at 310 K. SRB assay [45] was used to determine cell viability. Absorbance measurements of the solubilized dye allowed the determination of viable treated cells compared to untreated controls. IC_50_ values were determined as average values of triplicate experiments.

## 3 Results and Discussion

### 3.1 Synthesis and characterization of iridium complex Ir-I and Ir-II

**Scheme 1:**
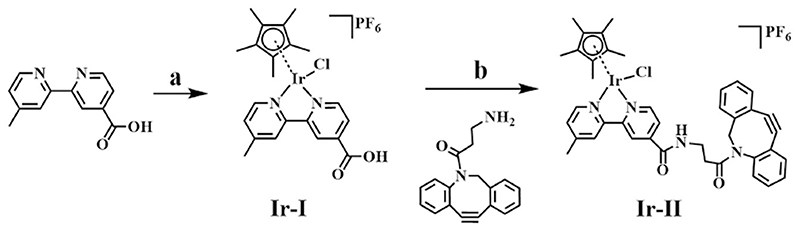
The synthetic route for iridium complex (Ir-II). Iridium complex Ir-I was prepared in step a and then subjected to amide coupling in step b to synthesize complex Ir-II. Reaction conditions (a): [(Cp*)Ir(μ-Cl)Cl]_2_, MeOH, 298 K, 18 h, then NH_4_PF_6_, 277 K, 18 h; (b): DBCO-NH_2_, oxalyl chloride, DCM, catalytic DMF, N_2_, 298 K, 2 h, then TEA, DCM, N_2_, 298 K, 4 h.

The half-sandwich iridium complex **(Ir-I)** was obtained by reacting the dimer [(Cp*)Ir(µ-Cl)Cl]2 with 4-methyl-4′-carboxy-2,2′-bipyridine,and subsequent anion exchange to a PF_6_- salt by addition of 10 molequiv. NH_4_PF_6_, shown as step a in Scheme 1. To synthesize complex **Ir-II**, the carboxylic acid group in **Ir-I** was then reacted with the amine group of DBCO-NH2 to afford **Ir-II**, a process facilitated by oxalyl chloride and the formation of a reactive acid chloride intermediate (step b in Scheme 1). Interestingly, the chloride ligand bound to the iridium survived under the amide reaction conditions. Both Ir-I and Ir-II were fully characterized by ^1^H NMR (Figs. S1, S3), ^13^C NMR (Figs. S2, S4) with peaks assigned, as well as high resolution ESI-MS in Figs. S5, S6. Half-sandwich iridium complex **Ir-I** containing a monodentate chloride ligand and an unsymmetrical N^N chelating ligand is chiral.Meanwhile, the eight-membered ring of the dibenzocyclooctyne moiety contains both enantiomers with R and S configuration of the amide nitrogen [46,47]. Thus, as a combination of above-mentioned two chiral components, complex **Ir-II** consists of two diastereomers with RS/SR and RR/SS configurations. The presence of two diastereomers were reflected by twin peaks with equal intensity in the UV–vis trace of HPLC corresponding to the same MS peak of *m/z* 835.2 (calcd *m/z* 835.2) assignable as **[Ir-II]** ^+^ (Fig. S6), and also manifested by double sets of peaks with ca. 1:1 ratio in the^1^H NMR and ^13^C NMR spectra of **Ir-II** (Figs. S3, S4).

### 3.2 X-ray crystal structure

**Fig. 1:**
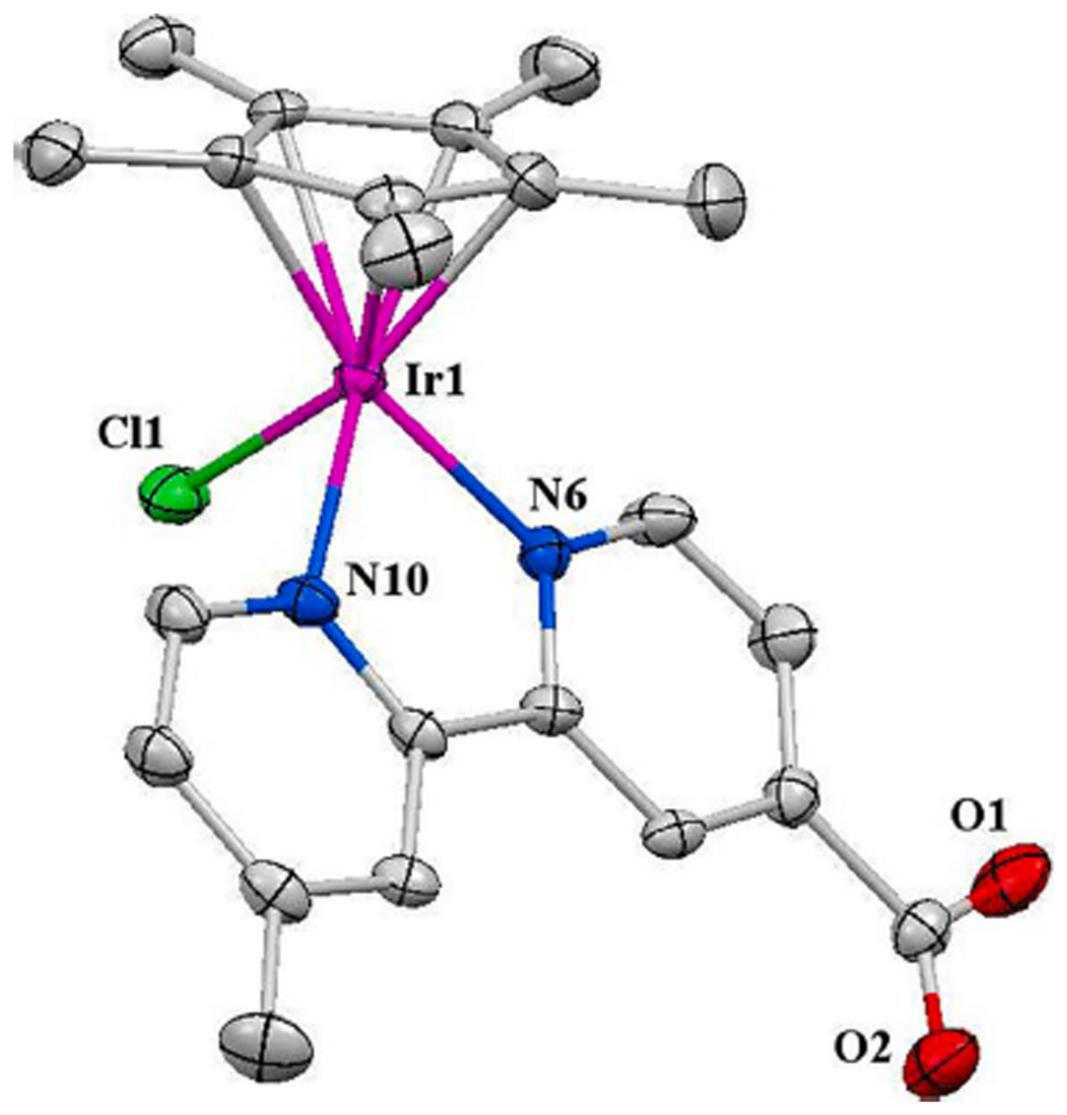
X-ray structure of [(Cp*)Ir(4-methyl-4′-carboxy-2,2′-bipyridine)Cl] PF_6_·MeOH (**Ir-I·MeOH**), with key atom labels, and drawn with thermal ellipsoids at 50% probability level. Hydrogen atoms and one methanol molecule and the PF_6_- counterion have been omitted for clarity.

Yellow block shaped crystals of **Ir-I** were obtained via the diffusion of diethyl ether into a saturated methanol solution at ambient temperature. Crystallographic data are shown in Table S1, and selected bond lengths and angles in Table S2. The structure with the key atoms numbered is shown in Fig. 1. The complex adopts the familiar threelegged “piano-stool” geometry. The Ir-N6 bond length of 2.088(4) Å is significantly shorter than the Ir-N10 bond length (2.096(4) Å). The distance between Ir and the centroid of the η^5^ -pentamethylcyclopentadienyl ring is 1.792 Å, and the Ir-Cl bond length 2.3952(14) Å. The distance between Ir and the centroid of the cyclopentadienyl ring is longer while the Ir-Cl bond is shorter than the corresponding lengths (1.786 and 2.404(2) Å, respectively) in complex [(Cp*)Ir(2,2’-bipyridine)Cl]Cl [48] suggesting that the deviations are due to the electronic effects from the methyl and carboxyl substituents.

### 3.3 Anticancer activity of Ir-I and Ir-II

The anticancer activity of iridium complexes **Ir-I** and **Ir-II** against A2780 human ovarian cancer cells was determined *in vitro* using the SRB assay after 24 h treatment and subsequent 72 h cell recovery. The IC_50_ value (half maximal inhibitory concentration) of complex **Ir-I** was >100 µM, showing that it is relatively non-toxic and inactive. However, complex **Ir-II** exhibited moderate anticancer activity against human ovarian cancer cells with IC50 value at 33.7 ± 0.3 µM compared to cisplatin (IC_50_ = 1.2 ± 0.1 µM).

In contrast, the ligand precursor DBCO-amine was relatively nontoxic (IC_50_ = 80 µM) at the IC_50_ concentration of complex **Ir-II** up to 24 h treatment. Similarly it is reported that a DBCO-dye (Cy5) conjugate is also non-toxic toward cancer cells [49]. The longer reversephase HPLC retention time for complex **Ir-II** (21.8 min) compared to the parent complex **Ir-I** (13.9 min, shown in Fig. S7) indicated that the addition of the DBCO group increased the lipophilicity of the resulting complex **Ir-II**, which is likely to lead to higher cellular iridium accumulation and subsequent higher cytotoxicity [15]. The carboxyl group on complex **Ir-I** will be deprotonated at (physiological) pH 7, giving a negative charge on the periphery of the complex, even though overall the complex will be neutral. In contrast, complex **Ir-II** has an overall positive charge, but no charge on the chelated bipyridyl ligand. Possible hydrolysis of these chloride complexes in the cell culture medium would also influence the speciation of the complex under screening conditions although high chloride concentrations are present (ca. 0.1 M) and may suppress hydrolysis, but this has yet to be studied.

### 3.4 Copper-free click synthesis of Ir-CP

**Fig. 2:**
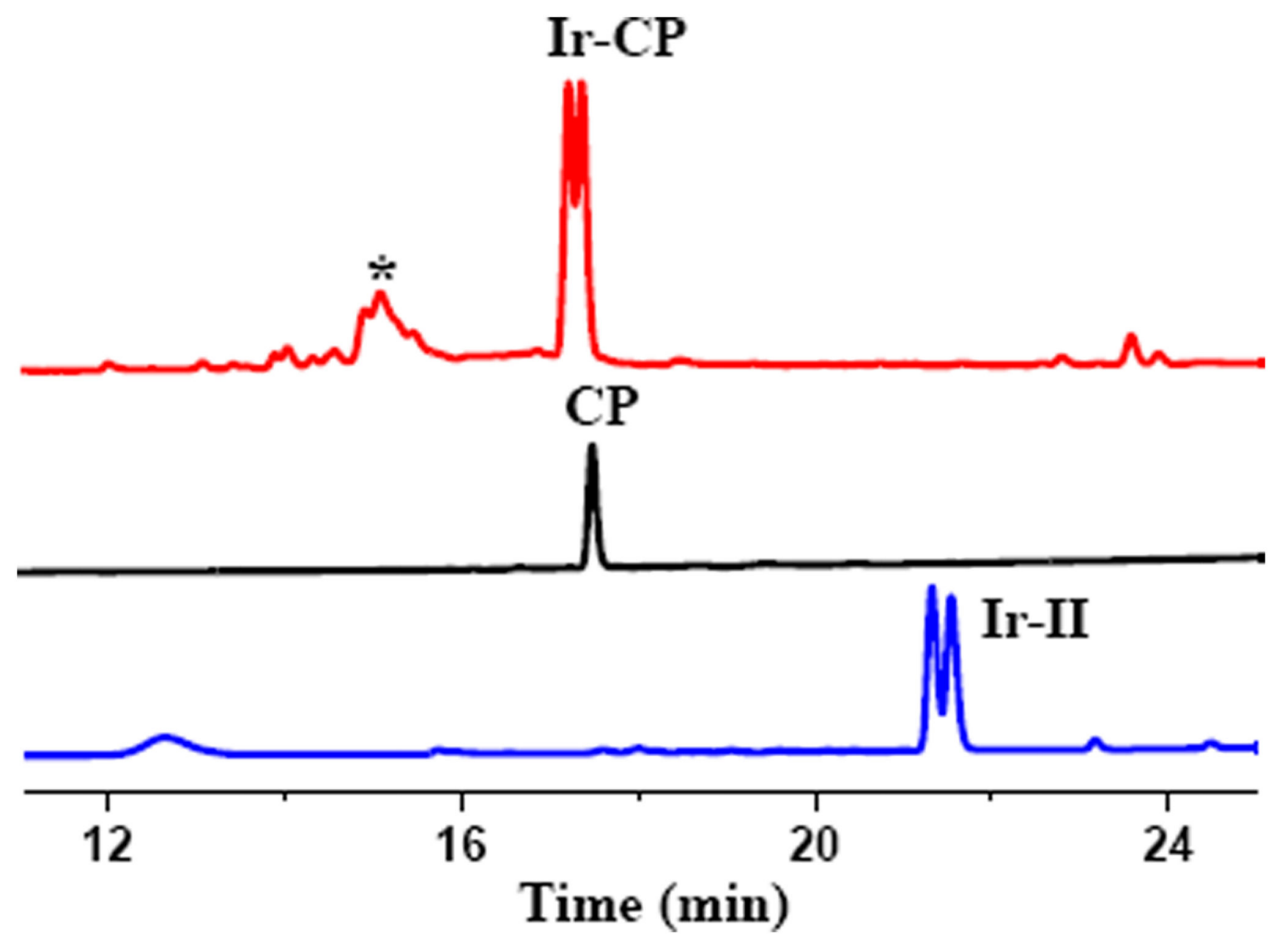
HPLC UV trace for cyclic peptide conjugate (**Ir-CP**), free peptide (**CP**) and **Ir-II** with all samples prepared in mixed 50 mM NaCl aqueous solution/acetonitrile (9/1, v/v). * denotes the aquated form of Ir-CP. Mobile phases: CH_3_CN/H_2_O with an additive of 0.1% v/v TFA.

**Scheme 2:**
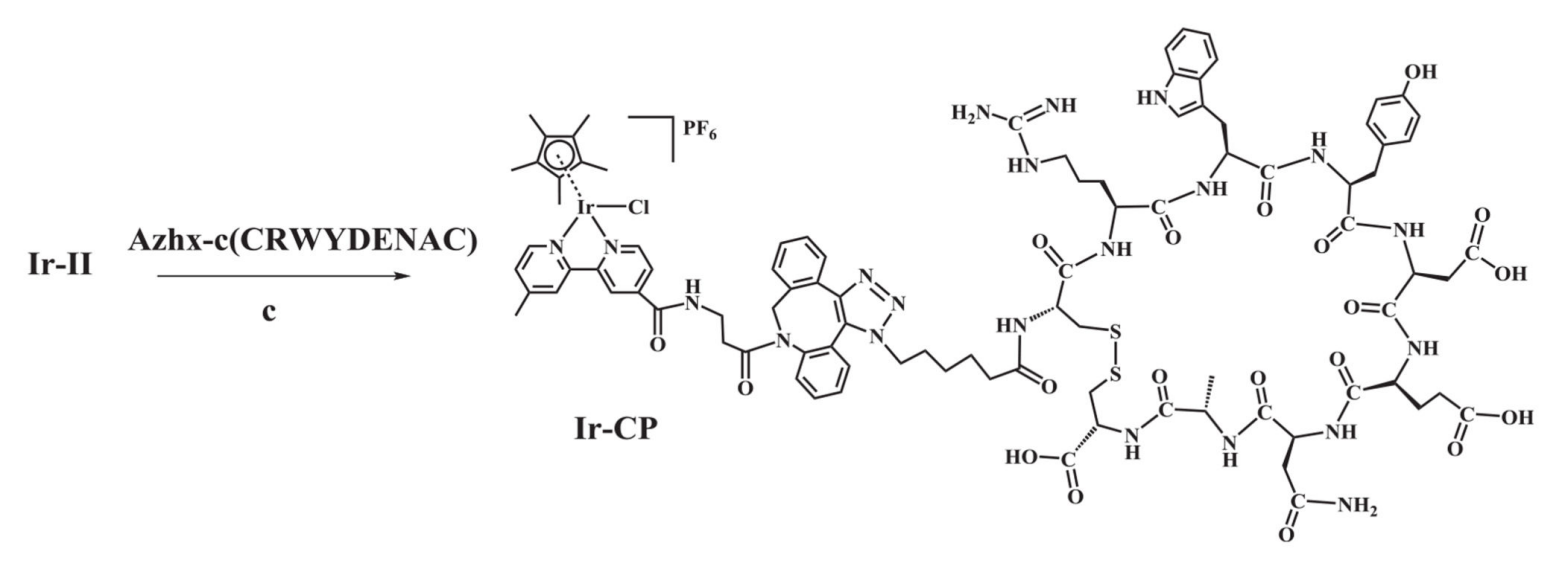
Synthetic route for the iridium-cyclic peptide conjugate complex (**Ir-CP**) via a copper-free click method under condition (c): DMF, N_2_, 298 K, 18 h.

The alkyne bond in the dibenzocyclooctyne group has been introduced as a specific reactive site in complex **Ir-II** for conjugation to the 6-azido hexanoic acid side chain of the modified cyclic peptide Azhx-c(CRWYDENAC). Synthesis of the peptide conjugate **Ir-CP** is facilitated by strain-promoted azide-alkyne cycloaddition of complex **Ir-II** to the cyclic peptide in DMF as shown in Scheme 2. The generation of Ir-CP by this copper-free click method was detected by HPLC analysis of the product. The **Ir-Cl** bonds of half-sandwich Cp* iridium chloride complexes with 2,2′-bipyridine ligands are often labile and readily hydrolyse in aqueous solution [15]. Therefore, 50 mM NaCl was added to the sample solution (acetonitrile and water, 1/9, v/v) for HPLC analysis to suppress the rapid hydrolysis of Ir-Cl bonds. The UV–vis trace of HPLC shown in Fig. 2 exhibited new twin peaks with retention times of ca. 17.5 min, distinct from the free cyclic peptide **(CP)** and precursor complex **Ir-II**. These new peaks were likely to belong to the peptide conjugate **Ir-CP**. In addition, given its twin peaks were of similar shape to those of the precursor complex **Ir-II**, **Ir-CP** also appeared to consist of diastereomers because of the diastereomeric reactant **Ir-II**. The fraction corresponding to the new twin peaks was collected and further analysed by LC-MS with unique *m*/*z* at 1065.54 (Fig. S8), assignable as [**(Ir-CP)** ^+H+^]^2+^ (calcd *m*/*z* 1065.38), which confirmed the formation of **Ir-CP**.

**Fig. 3:**
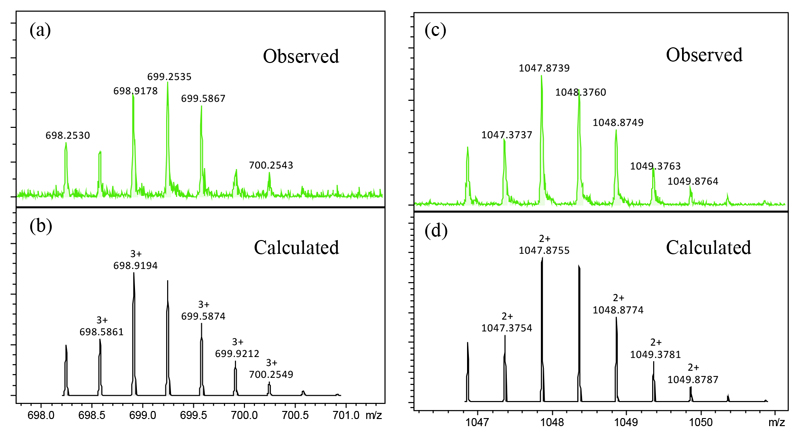
Observed and calculated high resolution mass spectra of the peptide conjugate in water/acetonitrile (9/1, v/v). (a) and (b) [**(Ir-CP)**-Cl)+H]^3+^; (c) and (d) [**(Ir-CP)**-Cl]^2+^.

In contrast, when sample of the **Ir-CP** for HPLC analysis was prepared in the absence of 50 mM NaCl, the peak with retention time of 15.2 min (Fig. S9) increased in intensity and was a major peak along with the previously mentioned peak at 17.5 min. This peak was further analysed by high resolution ESI-MS (Fig. 3), with *m/z* at 1047.8739, assignable as [**(Ir-CP)**-Cl]^2+^ (calcd *m/z* 1047.8755), and *m/z* at 698.9178 assignable as [**(Ir-CP)**-Cl+H]^3+^ (calcd *m/z* 698.9194). These data indicated that the peak at 15.2 min resulted from aquation of the Ir-Cl bond and the water molecule was lost under ESI-MS conditions.

**Fig. 4:**
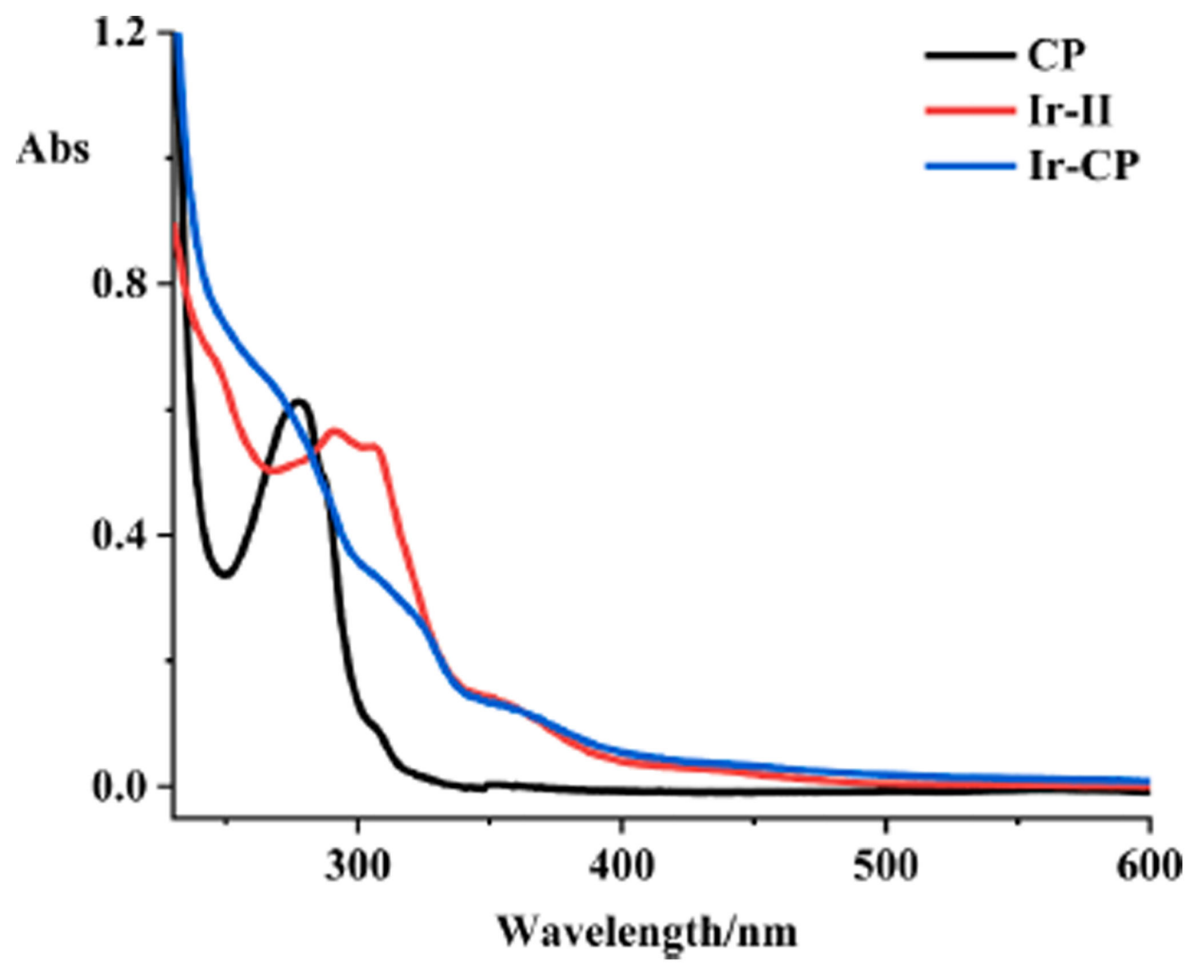
UV–vis absorption spectra of the peptide conjugate **Ir-CP**, the iridium precursor **Ir-II**, and the free cyclic peptide **CP** in PBS at 298 K.

The solubility of **Ir-CP** is higher than that of the precursor **Ir-II** in PBS, consistent with its shorter retention time at 17.5 min (lower hydrophobicity) compared to the retention time of **Ir-II** at 21.8 min (Fig. 2). The UV–vis absorption spectrum of **Ir-CP** was recorded in PBS at 298 K and compared with spectra of free peptide and **Ir-II**, as shown in Fig. 4. The free peptide **CP** showed a strong absorption at 278 nm, mainly due to the tyrosine and tryptophan residues [27]. **Ir-CP** not only retained the representative absorption of the **CP** at 278 nm, but also inherited the intense absorption band from precursor complex **Ir-II** at 300–320 nm due to π*-π*/n-π* transitions, with the band at 400–430 nm assigned as metal-to-ligand charge-transfer (MLCT) [50,51]. These characteristic features in the UV–vis spectrum of **Ir-CP** further illustrated the successful conjugation of the cyclic peptide to the iridium precursor.

## 4 Conclusions

Two novel half-sandwich iridium complexes **Ir-I** and the clickable analogue **Ir-II** have been synthesized and characterized by various analytical techniques, with their anticancer activity evaluated against the human ovarian cancer cell line. Significantly, the strain-promoted copper free azide-alkyne cycloaddition strategy has been utilized successfully to conjugate the half-sandwich iridium complex **Ir-II** to a tumour-targeting vector-azide-cyclic nonapeptide c(CRWYDENAC). The characterization of the peptide conjugate **Ir-CP** by HPLC, high resolution ESI-MS, as well as UV–vis spectroscopy has demonstrated the usefulness of this strategy for labelling organo-iridium complexes with targeting vectors. The moderate anticancer activity of the clickable complex **Ir-II** against ovarian cancer cells encourages investigation of the biological activity of the peptide conjugate in future work.

## 5 Author statement

All authors certify that they have participated sufficiently in the work to take public responsibility for the content, including participation in the concept, design, analysis, writing, or revision of the manuscript.

## Supplementary Material

**Appendix A. Supplementary data**

Supplementary data to this article can be found online at https://doi.org/10.1016/j.ica.2019.119396.

Supplementary data

## References

[1] Hanif M, Hartinger CG (2018). Future Med Chem.

[2] Kenny RG, Marmion CJ (2019). Chem Rev.

[3] Wang X, Wang X, Jin S, Muhammad N, Guo Z (2019). Chem Rev.

[4] Johnstone TC, Suntharalingam K, Lippard SJ (2016). Chem Rev.

[5] Barry NPE, Sadler PJ (2013). Chem Commun.

[6] Gasser G, Ott I, Metzler-Nolte N (2010). J Med Chem.

[7] Cutillas N, Yellol GS, de Haro C, Vicente C, Rodrfguez V, Ruiz J (2013). Coord Chem Rev.

[8] Zhang P, Sadler PJ (2017). J Organomet Chem.

[9] Ma D-L, Chan DS-H, Leung C-H (2014). Acc Chem Res.

[10] Geldmacher Y, Oleszak M, Sheldrick WS (2012). Inorg Chim Acta.

[11] Liu Z, Sadler PJ (2014). Acc Chem Res.

[12] Parveen S, Hanif M, Leung E, Tong KKH, Yang A, Astin J, De Zoysa GH, Steel TR, Goodman D, Movassaghi S, Sohnel T (2019). Chem Commun.

[13] Pontes da Costa A, Viciano M, Sanau M, Merino S, Tejeda J, Peris E, Royo B (2008). Organometallics.

[14] Kohl G, Pritzkow H, Enders M (2008). Eur J Inorg Chem.

[15] Liu Z, Habtemariam A, Pizarro AM, Fletcher SA, Kisova A, Vrana O, Salassa L, Bruijnincx PCA, Clarkson GJ, Brabec V, Sadler PJ (2011). J Med Chem.

[16] Soldevila-Barreda JJ, Metzler-Nolte N (2019). Chem Rev.

[17] Li J, Tian M, Tian Z, Zhang S, Yan C, Shao C, Liu Z (2018). Inorg Chem.

[18] Yang Y, Ge X, Guo L, Zhu T, Tian Z, Zhang H, Du Q, Peng H, Ma W, Liu Z (2019). Dalton Trans.

[19] Guo L, Zhang H, Tian M, Tian Z, Xu Y, Yang Y, Peng H, Liu P, Liu Z (2018). New J Chem.

[20] Li J, Guo L, Tian Z, Tian M, Zhang S, Xu K, Qian Y, Liu Z (2017). Dalton Trans.

[21] Ruiz J, Rodríguez V, Cutillas N, Samper KG, Capdevila M, Palacios Ò, Espinosa A (2012). Dalton Trans.

[22] Qiu K, Zhu H, Rees TW, Ji L, Zhang Q, Chao H (2019). Coord Chem Rev.

[23] Larnaudie SC, Brendel JC, Romero-Canelón I, Sanchez-Cano C, Catrouillet S, Sanchis J, Coverdale JP, Song J-I, Habtemariam A, Sadler PJ (2017). Biomacromolecules.

[24] Albada B, Metzler-Nolte N (2016). Chem Rev.

[25] Dissanayake S, Denny WA, Gamage S, Sarojini V (2017). J Controlled Release.

[26] Feng GK, Zhang MQ, Wang HX, Cai J, Chen SP, Wang Q, Gong J, Leong KW, Wang J, Zhang X (2019). Adv Therap.

[27] Shi H, Wang Q, Venkatesh V, Feng G, Young LS, Romero-Canelon I, Zeng M, Sadler PJ (2019). Dalton Trans.

[28] Krizek BA, Amann BT, Kilfoil VJ, Merkle DL, Berg JM (1991). J Am Chem Soc.

[29] Zou R, Wang Q, Wu J, Wu J, Schmuck C, Tian H (2015). Chem Soc Rev.

[30] Axup JY, Bajjuri KM, Ritland M, Hutchins BM, Kim CH, Kazane SA, Halder R, Forsyth JS, Santidrian AF, Stafin K (2012). Proc Natl Acad Sci USA.

[31] De Graaf AJ, Kooijman M, Hennink WE, Mastrobattista E (2009). Bioconjugate Chem.

[32] Hu Q-Y, Berti F, Adamo R (2016). Chem Soc Rev.

[33] Sletten EM, Bertozzi CR (2009). Angew Chem Int Ed.

[34] Lallana E, Riguera R, Fernandez-Megia E (2011). Angew Chem Int Ed.

[35] Thirumurugan P, Matosiuk D, Jozwiak K (2013). Chem Rev.

[36] Laughlin ST, Baskin JM, Amacher SL, Bertozzi CR (2008). Science.

[37] Poloukhtine AA, Mbua NE, Wolfert MA, Boons G-J, Popik VV (2009). J Am Chem Soc.

[38] Chang PV, Prescher JA, Sletten EM, Baskin JM, Miller IA, Agard NJ, Lo A, Bertozzi CR (2010). Proc Natl Acad Sci USA.

[39] McCafferty DG, Bishop BM, Wall CG, Hughes SG, Mecklenberg SL, Meyer TJ, Erickson BW (1995). Tetrahedron.

[40] Perry WS, Pope SJ, Allain C, Coe BJ, Kenwright AM, Faulkner S (2010). Dalton Trans.

[41] Tonnemann J, Risse J, Grote Z, Scopelliti R, Severin K (2013). Eur J Inorg Chem.

[42] Dolomanov OV, Bourhis LJ, Gildea RJ, Howard JA, Puschmann H (2009). J Appl Cryst.

[43] Sheldrick GM (2015). Acta Cryst.

[44] Sheldrick GM (2015). Acta Cryst.

[45] Vichai V, Kirtikara K (2016). Nat Protoc.

[46] Cruchter T, Harms K, Meggers E (2013). Chem Eur J.

[47] Jåger M, Gorls H, Giinther W, Schubert US (2013). Chem Eur J.

[48] Youinou M-T, Ziessel R (1989). J Organomet Chem.

[49] Kang S-W, Lee S, Na JH, Yoon HI, Lee D-E, Koo H, Cho YW, Kim SH, Jeong SY, Kwon IC, Choi K, Kim K (2014). Theranostics.

[50] Kalidasan M, Nagarajaprakash R, Forbes S, Mozharivskyj Y, Rao KM, Anorg Z (2015). Allg Chem.

[51] Palepu NR, Kaminsky W, Kolli para MR (2017). J Chem Sci.

