# Supplementary material for: Strategies for conjugating iridium(III) anticancer complexes to targeting peptides via copper-free click chemistry
Source: Inorganica Chim Acta. Author manuscript; Available in PMC 2021 Mar 27. (PMC7610455; doi:10.1016/j.ica.2019.119396)
Supplement: Supplementary data [file EMS119415-supplement-Supplementary_data.docx]

**Supporting information**

**Strategies for conjugating iridium(III) anticancer complexes to targeting peptides via copper-free click chemistry**

Wen-Ying Zhang^a^, Samya Banerjee^a^, Cinzia Imberti^a^, Guy J. Clarkson^a^, Qian Wang^b^, Qian Zhong^b^, Lawrence S. Young^c^, Isolda Romero-Canelón^a,d^, Musheng Zeng^b^, Abraha Habtemariam^a^, Peter J. Sadler^a,^*

^a^ Department of Chemistry, University of Warwick, Coventry CV4 7AL, UK

^b^ State Key Laboratory of Oncology in South China, Collaborative Innovation Center for Cancer Medicine, Sun Yat-Sen University Cancer Center, Guangzhou 510060, China

^c^ Medical School, University of Warwick, Coventry CV4 7AL, UK

^d^ School of Pharmacy, Institute of Clinical Sciences, University of Birmingham, Birmingham B15 2TT, UK

**Contents**

**Table S1.** Crystal data and refinement for complex **Ir-I·MeOH**.

**Table S2.** Selected bond length (Å) and angles (deg) for complex **Ir-I·MeOH**.

**Figures S1, S2.** ^1^H and ^13^C NMR spectra for **Ir-I** with peaks assigned.

**Figures S3, S4.** ^1^H and ^13^C NMR spectra for **Ir-II** with peaks assigned.

**Figures S5, S6.** High resolution ESI-MS analysis for **Ir-I** and **Ir-II**.

**Figure S7**. HPLC UV-vis trace of complex **Ir-I**.

**Figure S8.** ESI-MS of **Ir-CP**.

**Figure S9.** HPLC analysis of **Ir-CP** in the presence and absence of 50 mM NaCl.

**Table S1.** Crystal data and refinement for complex **Ir-I·MeOH**.

| Parameters | **Ir-I·MeOH** |
| --- | --- |
| Empirical formula | C_23_H_29_ClF_6_IrN_2_O_3_P |
| Formula weight | 754.10 |
| Temperature/K | 150(2) |
| Crystal system | monoclinic |
| Space group | P2_1_/n |
| *a*/Å | 8.0171(2) |
| *b*/Å | 15.9750(4) |
| *c*/Å | 21.1432(5) |
| *α*/° | 90 |
| *β*/° | 91.390(2) |
| *γ*/° | 90 |
| Volume/Å^3^ | 2707.09(12) |
| Z | 4 |
| ρ_calc_g/cm^3^ | 1.850 |
| *μ*/mm^‑1^ | 5.159 |
| F(000) | 1472.0 |
| Crystal size/mm^3^ | 0.16 × 0.04 × 0.04 |
| Radiation | MoKα (λ = 0.71073) |
| 2*Θ* range for data collection/° | 5.392 to 62.114 |
| Index ranges | -11 ≤ h ≤ 10,  -21 ≤ k ≤ 22,  -30 ≤ l ≤ 30 |
| Reflections collected | 35859 |
| Independent reflections | 7851 [R_int_ = 0.0464, R_sigma_ = 0.0470] |
| Data/restraints/parameters | 7851/0/343 |
| Goodness-of-fit on F^2^ | 1.116 |
| Final R indexes [I>=2σ (I)] | R_1_ = 0.0452, wR_2_ = 0.0853 |
| Final R indexes [all data] | R_1_ = 0.0582, wR_2_ = 0.0898 |
| Largest diff. peak/hole/e Å^-3^ | 2.31/-1.54 |

**Table S2.** Selected bond length (Å) and angles (deg) for complex **Ir-I·MeOH**.

| **Bond(s)** | **Ir-I·MeOH** |
| --- | --- |
| Ir-C (Cp^*^) | 2.201(5) |
|  | 2.160(5) |
|  | 2.151(5) |
|  | 2.158(5) |
|  | 2.173(5) |
| Ir-C (Centroid) | 1.792 |
| Ir-N6 (opposite to -COOH) | 2.088(4) |
| Ir-N10 (opposite to -CH_3_) | 2.096(4) |
| Ir-Cl | 2.3952(14) |
| N6-Ir-N10 | 76.18(16) |
| N6-Ir-Cl | 86.99(12) |
| N10-Ir-Cl | 86.88(12) |

**Figure S1.** ^1^H NMR (400 MHz, 298 K) spectrum of **Ir-I** in d_4_-MeOD with peaks assigned.

**Figure S2.** ^13^C APT NMR (125 MHz, 298 K) spectrum of **Ir-I** in d_4_-MeOD with peaks assigned.

**Figure S3.** ^1^H NMR (400 MHz, 298 K) spectrum of **Ir-II** in d_4_-MeOD with peaks assigned to the two diastereomers numbered without and with superscript’.

**Figure S4.** ^13^C APT NMR (125 MHz, 298 K) spectrum of **Ir-II** in d_4_-MeOD with peaks assigned.

**Figure S5.** Experimental (upper) and calculated (lower) high resolution ESI mass spectra of **Ir-I** for the molecular ion [(**Ir-I**-H)+Na]^+^.

**Figure S6**. Experimental (upper) and calculated (lower) high resolution ESI mass spectra of **Ir-II** for the molecular ion [(**Ir-II)**-PF_6_]^+^.


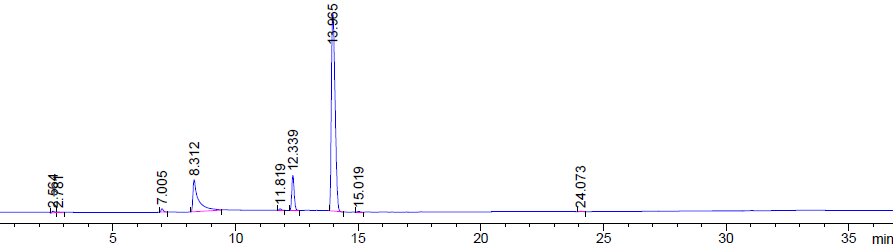


**Figure S7**. HPLC UV-vis trace for complex **Ir-I** in acetonitrile/water (1/9 v/v). The peak with retention time of 13.9 min corresponds to [(**Ir-I**)-PF_6_]^+^. Mobile phases: ACN/H_2_O (0.1% v/v TFA), detection wavelength 254 nm.

**Figure S8.** LC-MS ESI-MS detection at 17.5 min with *m/z* 1065.54, assignable as [(**Ir-CP**)+H^+^]^2+^ (calcd *m/z* 1065.38). Mobile phases: ACN/H_2_O (0.1% v/v TFA), detection wavelength 254 nm.

**Figure S9.** HPLC analysis of the peptide conjugate **Ir-CP** (100 µM) prepared in acetonitrile/water (1/9 v/v) (a) without NaCl; (b) with 50 mM NaCl at 298 K. Peak **1** corresponds to the peptide conjugate [(**Ir-CP**)+H^+^]^2+^; peak **2** corresponds to peptide conjugate [(**Ir-CP**)-Cl]^2+^ resulting from aquation of the Ir-Cl bond with the water molecule lost under ESI-MS conditions. Mobile phases: ACN/H_2_O (0.1% v/v TFA), detection wavelength 254 nm.
